# Case report: A case of neoadjuvant immunotherapy in combination with the Yang–Monti technique for the treatment of ureteral carcinoma after radical cystectomy and left radical nephroureterectomy

**DOI:** 10.3389/fonc.2022.889028

**Published:** 2022-07-28

**Authors:** Tielin Wu, Harris Haleem, Min Yin

**Affiliations:** Department of Urology, Ningbo Medical Treatment Centre Li Huili Hospital, Ningbo, China

**Keywords:** neoadjuvant immunotherapy, tislelizumab, Yang-Monti technique, solitary kidney, Recurrence of urothelial carcinoma, anti-programmed death protein-1

## Abstract

Recurrence of urothelial carcinoma in a patient with solitary kidney is always a clinical challenge. In the immune checkpoint inhibitor era, neoadjuvant immunotherapy in combination with the Yang–Monti technique might be a good option for the patient with a high-risk tumor when kidney-sparing surgery for renal function preservation is desired. We report the case of a 74-year-old man with solitary kidney who was diagnosed with recurrence of urothelial carcinoma in the right ureter. He was initially deemed unfit for segmental resection of the ureter. Neoadjuvant immunotherapy with tislelizumab was performed in this patient with a partial response to urothelial carcinoma. He underwent segmental resection of the ureter with negative margins, and the ureteral defect was bridged by modified ileal replacement, which is the Yang–Monti technique. This patient has remained disease-free with adequate kidney function for longer than 18 months.

## Background

The upper urinary tract is the most common site of late recurrence following bladder cancer (1). Recurrence of urothelial carcinoma in a patient with solitary kidney is always a clinical challenge. In low-risk cancers, kidney-sparing surgery (KSS) is the preferred approach as survival is similar to that after radical nephroureterectomy (RNU),which includes removing the kidney, ureter, and partially the bladder (2). However, it is still unknown what is the best choice for high-risk tumors when KSS for renal function preservation is desired. Seisen showed that there was no difference in cancer-specific survival or other oncologic outcomes between KSS and RNU; thus, segmental ureterectomy could also be used in a select subset of high-grade and invasive UTUC (2). The guidelines recommend case-dependent consideration of biopsy grade, imaging findings, and urine cytology when deciding between RNU and KSS (1). After segmental ureterectomy, long ureteral defects remained a challenge to urologists. Many techniques, such as ileal ureter, appendiceal interposition, and reconfigured colon substitution, have been described in literature to reconstruct the long defection of ureter rather than direct re-anastomosis. The Yang–Monti principle, which was firstly described by Yang in 1993 and was verified by Monti et al., allows the creation of a long tube from a short bowel segment after its reconfiguration (3, 4).

Neoadjuvant platinum-based chemotherapy represents the standard treatment for non-metastatic muscle invasive bladder cancer (MIBC) (5). However, there are cisplatin-ineligible patients, who could not benefit from chemotherapy before surgery. PURE-01 was a neoadjuvant immunotherapy trial for bladder cancer, which had shown a pathological complete response (pT0) rate of 42%, and pT0 was associated with high tumor mutational burden (TMB) and DNA damage response (DDR) gene alterations (6). Despite the good outcomes in MIBC, neoadjuvant therapy in UTUC does not yield level 1 evidence as there are only several retrospective studies published thus far. Tislelizumab, an anti-programmed death protein-1 (PD-1) monoclonal antibody, demonstrated meaningful clinical benefits in patients with previously treated locally advanced urothelial carcinoma and had a manageable safety profile (7). We report a case of recurrence of urothelial carcinoma with DDR gene alterations and high TMB, which got a partial response to neoadjuvant immunotherapy with tislelizumab. After neoadjuvant immunotherapy, segmental ureterectomy was performed and a long ureteral defect was bridged with the Yang–Monti technique.

## Case presentation

**Figure 1:**
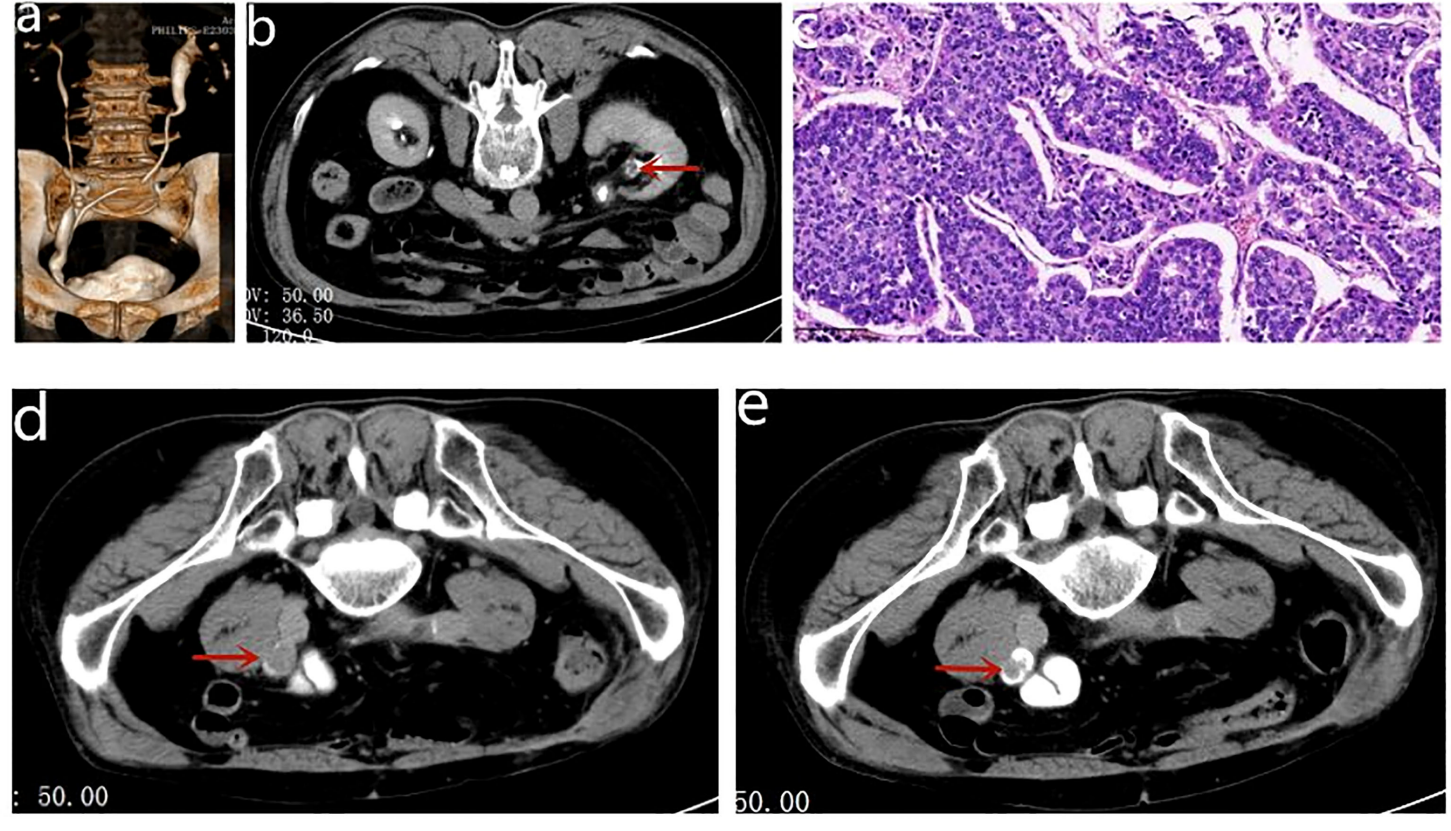
**(A)** CTU revealed that orthotopic neobladder was in good shape and there was no hydronephrosis in both renal pelvis. **(B)** The red arrows indicate the morphological changes in the left renal pelvis. **(C)** The pathological result identified high-grade urothelial papillary carcinoma of the left renal pelvis. **(D)** CT revealed recurrence of the right ureteral carcinoma with obstructive hydronephrosis. **(E)** CT revealed that the maximum diameter of the tumor shrank from 25 to 15 mm.

This case report was approved by the research ethics committee of Ningbo Medical Treatment Centre Li Huili Hospital (Ethical Approval Number: QT2020PJ050). A 74-year-old man who had a history of radical cystectomy and left radical nephroureterectomy due to MIBC and left renal pelvis carcinoma was diagnosed with recurrent urothelial carcinoma in the right ureter. In 2012, this patient was diagnosed with MIBC and received laparoscopic radical cystectomy + pelvic lymph node dissection (PLND) accompanied by orthotopic neobladder reconstruction with Ileal conduit, pathologically staged as T2N0M0. The postoperative pathology was high-grade invasive urothelial carcinoma that was partly micropapillary and showed infiltration into the lamina propria; no metastasis was seen in 17 retrieved lymph nodes. He was treated with adjuvant cisplatin-containing combination chemotherapy for four courses. Patient follow-up was performed from 2012 to 2018 with abdominal-pelvis computed tomography (CT) (**Figure 1A**). In 2019, 7 years after radical cystectomy, he showed macrohematuria. CT showed morphological changes in the left renal pelvis without a ureteral lesion present (**Figure 1B**), and cancer cells were found in urine cytology. Radical nephroureterectomy was performed in April 2019. The left kidney and ureter along with a part of bladder were removed. The pathological result identified high-grade urothelial papillary carcinoma of the left renal pelvis, with a mass size of 3 cm * 2 cm *1 cm, infiltrating the fat of the renal sinus and close to the renal parenchyma, involving the proximal ureter, with visible choroidal carcinoma thrombus. The lesion was indicated as locally advanced urothelial carcinoma with a stage of T3N0M0 (**Figure 1C**).

**Figure 2:**
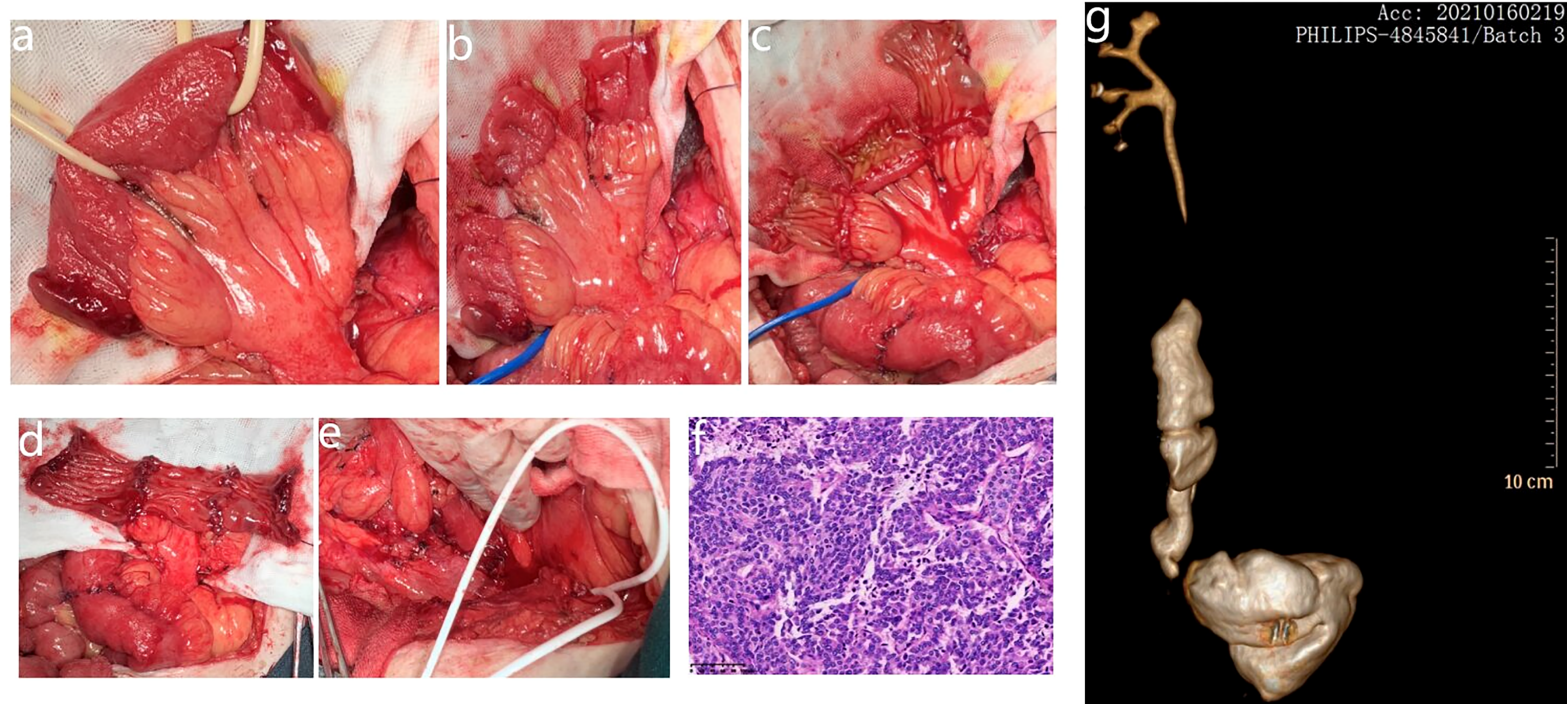
**(A)** The intestinal substitute is derived from the ileum. **(B)** Intestinal sections isolated with their mesenteric branch. The continuity of the small intestine is restored. **(C)** Each ring is then incised along its longitudinal axis; the incisions of the most proximal and distal segments are not at the antimesenteric border but close to the mesenteric attachments. **(D)** Intestinal strips joined end to end. **(E)** The tissue plate tubularized around a single “J” stent to form the neoureter. **(F)** The postoperative pathology was invasive high-grade urothelial carcinoma. **(G)** CTU revealed that the neoureter was in good shape and that there was no hydronephrosis in the right renal pelvis.

**Figure 3:**
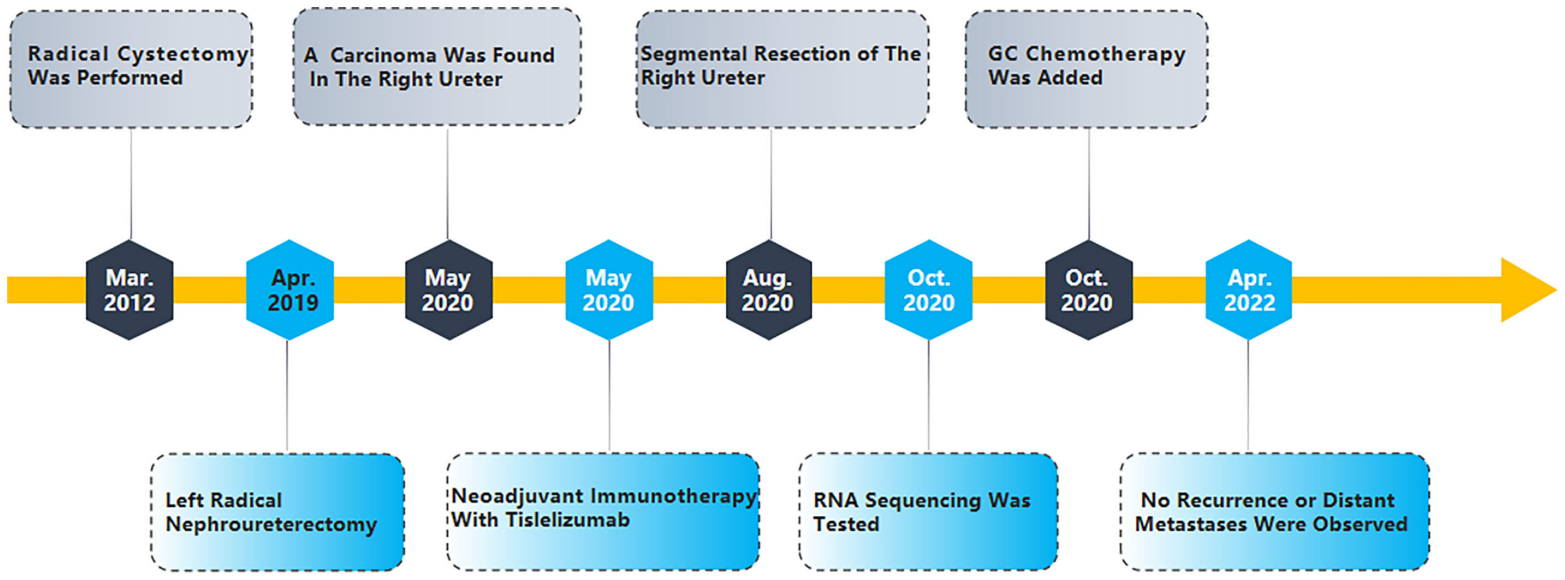
Treatment Timeline.

After 1 year of regular follow-up, CT revealed recurrence of the right ureteral carcinoma with obstructive hydronephrosis, cT3N0M0 (**Figure 1D**). The calculated eGFR was 37.157 ml/min/1.73 m^2^; considering the risk of renal failure due to cisplatin, the patient was treated with tislelizumab (200 mg ivgtt q3w) for three courses. Nine weeks later, CT revealed that the maximum diameter of the tumor shrank from 25 to 15 mm, suggesting a partial response (PR) to tislelizumab (**Figure 1E**). This patient had a strong desire to preserve his kidney and refused to receive a right ureteronephrectomy associated with hematodialysis. Therefore, he was treated with partial right ureteral resection (including part of the input collaterals of the original Studer bladder) + modified ileal replacement for ureteral defect, which is the Yang–Monti technique. Firstly, the right ureteral tumor was removed and then a section of approximately 12 cm of ileum was isolated on its mesenteric branch. The isolated ileal segment is then divided into three ileal pieces accompanying mesenteric blood supply. These rings are opened with an incision at the paramesenteric border. These three strips are joined at the ends, to make a rectangular piece of 18 cm in length, which is then tubularized around a single “J” stent to form a tube of suitable length (**Figures 2A-E**). The operation went well, and the postoperative pathology was invasive high-grade urothelial carcinoma, with infiltration into the muscular layer of the canal wall and negative margins on both sides (**Figure 2F**). Five days after the operation, the patient began to develop psychiatric symptoms such as prosopagnosia. We speculated that the psychiatric symptoms might be caused by postoperative stress; thus, the patient received sedation and oral symptomatic treatment with risperidone. The status of the patient gradually improved and he was discharged in about 10 days. After discharge, he received continuous tislelizumab treatment. After 2 months of follow-up, his calculated eGFR was 53.71 ml/min/1.73 m^2^, indicating the recovery of renal function. RNA sequencing with DDR gene alterations showed a possible beneficial role of platinum-based chemotherapy. The patient completed four courses of GC chemotherapy in combination with tislelizumab immunotherapy. He is still on tislelizumab immunotherapy at 18 months of follow-up, and no recurrence or distant metastases were observed (**Figure 2G**). The treatment flowchart is shown in **Figure 3**.

## Discussion

We describe a patient who initially presented with recurrence of urothelial carcinoma in the ureter after radical cystectomy and left radical nephroureterectomy. He was started on neoadjuvant immunotherapy with tislelizumab and had a partial pathologic response within the tumor. Tislelizumab was well-tolerated and resulted in vast improvement in performance status, which permitted consolidative surgical therapy with curative intent. As one type of ICIs, tislelizumab has been used to treat urothelial carcinoma. In our case, after three courses, tislelizumab shrank the tumor in both extension and diameter, downstaging clinically from cT3 to cT2. To investigate why this patient had such a good response to tislelizumab, RNA sequencing was tested, which suggested high TMB (31.84 Muts/Mb) and DDR gene alterations. High TMB and DDR gene alterations may suggest a good response to ICIs (6). Meanwhile, Min Yuen Teo showed that defects in DDR genes have a significant impact on sensitivity to platinum therapy (8). The POUT trial was a phase 3, open-label, randomized controlled trial, which had shown gemcitabine–platinum combination chemotherapy initiated within 90 days after nephroureterectomy significantly improved disease-free survival in patients with locally advanced UTUC (9).

To our knowledge, this is the first reported case of recurrence of urothelial carcinoma after radical cystectomy and left radical nephroureterectomy treated with neoadjuvant immunotherapy in combination with segmental ureterectomy, which was reconstructed by the Yang–Monti technique. This patient has a past history of MIBC 7 years ago, and has a recurrence of urothelial carcinoma in his right ureter less than 1 year after his treatment of left renal pelvis cancer. Meanwhile, CT showed that the tumor had invaded the fat around the ureter, suggesting a clinical stage cT3, which means that if this patient had a strong desire to preserve his kidney, neoadjuvant therapy was needed. However, the calculated eGFR was 37.157 ml/min/1.73 m^2^, and considering the high risk of renal failure due to cisplatin, neoadjuvant immunotherapy was worth trying. Fortunately, the tumor had a good response to tislelizumab, which made segmental ureterectomy plus the Yang–Monti technique a better option.

As shown by some investigators, the Yang–Monti technique is effective and has numerous advantages over both intact and tapering ileal ureters. It allows the reconfiguration of short ileal segments into long tubes of small caliber by converting the circular fibers into longitudinal ones (10). An ileal segment can replace approximately threefold length of ureteral defects after reconfiguration, and its diameter is close to that of the ureter. Yang–Monti ileal ureters mimic the function of a native ureter, providing active antegrade propulsion for urinary drainage (11). The possibility of excessive mucus production and metabolic acidosis is almost absent because of the reduction of surface area with secretive and absorptive characteristics (10). A nonconcurrent cohort study, in which Yang–Monti ileal ureter reconstruction was performed in 36 patients between 2001 and 2019, shows that the Yang–Monti ileal ureter is durable and effective in improving kidney function with few complications. It can be safely used in cases of mild/moderate kidney function loss and solitary kidney (12). Thus, because of these excellent results, the Yang–Monti technique gained wider recognition and acceptance.

There are some limitations to this analysis. Primarily, there are currently no approved neoadjuvant systemic treatments for patients with UTUC. Although, in our case, the urothelial carcinoma had a response to neoadjuvant immunotherapy, more cases still need to be explored to determine whether neoadjuvant immunotherapy could improve the OS. Secondly, the follow-up time is only 18 months; thus, we do not know whether the urothelial carcinoma is really curable.

## Conclusion

Preoperative immunotherapy with tislelizumab for locally advanced ureteral carcinoma accompanied by solitary kidney resulted in a partial response, which enabled curative intent and segmental resection of ureter. The Yang–Monti technique is an efficient way to correct clinical ureteral defects after segmental ureterectomy.

## Data Availability Statement

The original contributions presented in the study are included in the article/supplementary material. Further inquiries can be directed to the corresponding author.

## Ethics Statement

The studies involving human participants were reviewed and approved by The Research Ethics Committee of Ningbo Medical Treatment Centre Li Huili Hospital. The patients/participants provided their written informed consent to participate in this study.

## Author Contributions

TW and HH drafted the manuscript. MY did the surgery. All authors read and approved the final manuscript. All authors contributed to the article and approved the submitted version.

## Conflict of Interest

The authors declare that the research was conducted in the absence of any commercial or financial relationships that could be construed as a potential conflict of interest.

## Publisher’s Note

All claims expressed in this article are solely those of the authors and do not necessarily represent those of their affiliated organizations, or those of the publisher, the editors and the reviewers. Any product that may be evaluated in this article, or claim that may be made by its manufacturer, is not guaranteed or endorsed by the publisher.
